# Raloxifene Is Associated with Total Knee Arthroplasty in Postmenopausal Women: A Comparative Cohort Study

**DOI:** 10.3390/life15101531

**Published:** 2025-09-29

**Authors:** Jer-Yung Chen, Wen-Tien Wu, Ru-Ping Lee, Ting-Kuo Yao, Cheng-Huan Peng, Hao-Wen Chen, Jen-Hung Wang, Kuang-Ting Yeh

**Affiliations:** 1 School of Medicine, Tzu Chi University, Hualien 970374, Taiwan; 11311147@gms.tcu.edu.tw (J.-Y.C.);; 2 Department of Orthopedics, Hualien Tzu Chi Hospital, Buddhist Tzu Chi Medical Foundation, Hualien 970473, Taiwan; 3 Institute of Medical Sciences, Tzu Chi University, Hualien 970374, Taiwan; 4 Department of Medical Research, Hualien Tzu Chi Hospital, Buddhist Tzu Chi Medical Foundation, Hualien 970473, Taiwan; 5 Graduate Institute of Clinical Pharmacy, Tzu Chi University, Hualien 970374, Taiwan

**Keywords:** post-traumatic knee osteoarthritis, raloxifene, total knee replacement, postmenopausal women, anti-osteoporotic medications, bisphosphonates, denosumab

## Abstract

Post-traumatic osteoarthritis (PTOA) is a distinct form of knee osteoarthritis characterized by accelerated joint degeneration following injury. It poses unique challenges in post-menopausal women due to hormonal changes and altered bone metabolism that create complex pathophysiological environments. This retrospective cohort study compared the effectiveness of different anti-osteoporotic medications in preventing total knee replacement (TKR) in 6155 postmenopausal women with PTOA treated between 2011 and 2021. We compared raloxifene and denosumab with bisphosphonates serving as the reference group. The primary outcome was TKR occurrence. Cox proportional hazards regression and inverse probability of treatment weighting (IPTW) were performed to estimate hazard ratios, with Kaplan–Meier survival analysis for time-to-event assessment. Participants’ mean (SD) age was 69.4 (10.0) years. Given the retrospective nature and typical delayed presentation of PTOA symptoms, cohort entry was defined as the concurrent diagnosis of PTOA and osteoporosis requiring anti-resorptive therapy. Over a mean follow-up of 5.47 years, 99 patients (1.6%) underwent TKR. Raloxifene was associated with a significantly reduced TKR risk compared to bisphosphonates (IPTW-HR 0.81, 95% CI 0.67–0.99, *p* = 0.040), representing a 19% relative risk reduction. Kaplan–Meier analysis demonstrated raloxifene maintained the lowest cumulative TKR incidence compared to bisphosphonates and denosumab over time, particularly beyond 5 years. These findings suggest that raloxifene may offer superior joint protection compared with bisphosphonates and denosumab in postmenopausal women with PTOA, supporting its potential as a disease-modifying therapeutic option for this vulnerable population.

## 1. Introduction

Knee osteoarthritis (OA) is a leading cause of chronic pain and disability worldwide [1,2] and a common degenerative joint disease with radiographic evidence present in over 30% of adults aged ≥ 65 years [3]. This rising prevalence of OA is reflected in the increasing demand for total knee replacement (TKR) for end-stage cases [1,3]. Real-world data from Taiwan show that, in older women with both knee OA and osteoporosis (OP), the choice of anti-resorptive therapy (denosumab vs. bisphosphonates) may influence the eventual need for TKR [4]. Importantly, OA disproportionately affects women—especially postmenopausal women—who experience higher disease prevalence, more severe pain, faster structural progression, and lower surgical intervention rates compared to men [5]. These findings highlight a dual clinical challenge: while TKR is the definitive solution for advanced OA when conservative measures fail, coexisting osteoporosis compromises implant fixation and long-term prosthetic survival, making pharmacotherapeutic decisions crucial before and after surgery.

Posttraumatic osteoarthritis (PTOA) is a distinct form of knee OA characterized by accelerated joint degeneration following acute injury or repetitive microtrauma. In postmenopausal women, hormonal changes, altered bone metabolism, and mechanical joint compromise combine to create a complex pathophysiological environment that may respond differently to various anti-osteoporotic treatments. Anterior cruciate ligament (ACL) injury—more common in women—is a leading pathway to knee PTOA and contributes to the long-term osteoarthritis burden observed later in life. Understanding the comparative effectiveness of different anti-osteoporotic medications in this population is crucial to optimize treatment strategies and potentially delay the need for surgical intervention.

At the tissue level, osteoarthritis and osteoporosis share overlapping pathogenic features associated with skeletal aging, including senescent osteoclast accumulation in the subchondral region that accelerates bone turnover and creates a mechanically unfavorable environment promoting cartilage degeneration [6,7,8,9,10]. Preclinical evidence demonstrates that selective estrogen receptor modulators (SERMs) like raloxifene can suppress TGF-β1 over-expression in cartilage, normalize subchondral bone metabolism, preserve type II collagen, reduce matrix metalloproteinase activity, and protect the osteochondral interface from mechanical injury [11,12,13,14]. Despite this mechanistic rationale, clinical evidence remains inconsistent: a 2024 systematic review found mixed effects of anti-resorptives on OA structure and symptoms with no consensus that they delay or prevent TKR, and real-world uptake of osteoporosis therapy remains suboptimal [15,16,17,18,19].

The comparative effectiveness of different anti-osteoporotic medications in preventing TKR progression among postmenopausal women with PTOA remains poorly characterized in clinical practice. Although bisphosphonates are the most prescribed first-line therapies for osteoporosis, denosumab offers a more potent anti-resorptive mechanism through RANKL inhibition, and raloxifene provides unique estrogen receptor-mediated effects on both bone and cartilage metabolism. Given their distinct mechanisms of action and the complex interplay between bone and cartilage homeostasis in PTOA, a comprehensive comparison of these therapeutic approaches is essential to inform evidence-based clinical decision-making.

To address this gap, we conducted a retrospective cohort study of 6155 post-menopausal women with post-traumatic knee OA treated with raloxifene, denosumab, or bisphosphonates between 2011 and 2021 at a major medical center in Taiwan. We hypothesized that raloxifene—through estrogen-receptor mediated modulation of subchondral bone and articular cartilage—would confer superior protection against progression to TKR compared to other anti-resorptive therapies. This study aimed to provide real-world evidence on the comparative effectiveness of three major classes of anti-osteoporotic medications in delaying the need for TKR surgery in this vulnerable population.

## 2. Materials and Methods

This retrospective cohort study was conducted using data from the electronic medical records database of Hualien Tzu Chi Hospital, covering the period from January 2011 to December 2021. The study protocol was approved by the Ethics Research Committee of Hualien Tzu Chi Hospital (approval number: IRB112-133C), and the requirement for informed consent was waived due to the retrospective nature of the study. All procedures adhered to the ethical standards of the Institutional Research Committee and the principles of the 1964 Declaration of Helsinki and its subsequent amendments. We identified postmenopausal women diagnosed with PTOA of the knee who had received anti-osteoporotic medication for at least 1 year. PTOA was defined based on the International Classification of Diseases, 10th Revision (ICD-10) codes M17.2 and M17.3, with documented evidence of knee trauma in the medical history. Given the retrospective nature and the typical delay between initial trauma and symptomatic PTOA presentation in postmenopausal women, we defined the cohort entry point as the first concurrent diagnosis of PTOA and osteoporosis requiring anti-resorptive therapy, rather than attempting to trace back to the original traumatic event. Postmenopausal status was determined by either natural menopause (absence of menstruation for at least 12 consecutive months) or surgical menopause (bilateral oophorectomy), as recorded in the medical history. The study period (2011–2021) was selected to ensure electronic medical record completeness and standardization, capture the era of routine availability of all three anti-osteoporotic medications, and align with consistent ICD-10 coding implementation at our institution. We specifically focused on PTOA rather than all knee osteoarthritis for several methodological and clinical reasons. First, PTOA represents a more homogeneous disease entity with a clear precipitating event, reducing confounding from varied osteoarthritis etiologies including primary, metabolic, and inflammatory causes. Second, PTOA patients are typically younger at disease onset compared to primary osteoarthritis, providing a more suitable population for studying long-term anti-osteoporotic medication effects over extended follow-up periods. Third, PTOA has distinct inflammatory and metabolic characteristics that may respond differently to anti-osteoporotic medications compared to primary osteoarthritis, given the unique pathophysiological cascade following joint trauma. Finally, PTOA represents a significant clinical burden, particularly in active populations, and understanding optimal treatment strategies is crucial for this specific patient group where prevention of TKR progression may have substantial quality-of-life implications.

The index date was defined as the initiation of anti-osteoporotic therapy following the concurrent diagnosis of PTOA and osteoporosis. This approach ensures a clinically relevant starting point while maintaining comparability between treatment groups. The inclusion criteria were (1) postmenopausal women aged 50 years or older; (2) first-time diagnosis of PTOA of the knee with documented evidence of previous traumatic knee injury in the medical history, defined as acute knee trauma including ligamentous injuries, meniscal tears, fractures involving the knee joint, or significant contusions that preceded the PTOA diagnosis; (3) concurrent diagnosis of osteoporosis based on bone mineral density testing (T-score ≤ −2.5) or clinical risk factors; (4) treatment with one of the following anti-osteoporotic medications for at least 1 year: bisphosphonates (alendronate, risedronate, ibandronate, or zoledronic acid), denosumab, or raloxifene; and (5) regular follow-up visits with complete medical records. Exclusion criteria were (1) history of inflammatory arthritis, including rheumatoid arthritis, psoriatic arthritis, or ankylosing spondylitis; (2) previous TKR or other knee surgeries before the index date; (3) chronic kidney disease stage 4 or 5 (estimated glomerular filtration rate < 30 mL/min/1.73 m^2^); (4) cognitive disorders affecting medication adherence or follow-up; (5) malignancy requiring chemotherapy or radiotherapy; (6) concurrent use of multiple anti-osteoporotic medications; (7) treatment duration less than 1 year with the index medication; and (8) concurrent hormone replacement therapy.

Data were extracted by trained research assistants using a standardized data collection form. Demographic data, including age at index date was recorded. Comorbidities were identified using ICD-10 codes and included hypertension (I10–I15), diabetes mellitus (E10–E14), dyslipidemia (E78), liver disease (K70–K77), cerebrovascular disease (I60–I69), and coronary artery disease (I20–I25). Patients were categorized into three groups based on the anti-osteoporotic medications received: bisphosphonate, denosumab, and raloxifene. For patients who switched medications during the study period, only the first medication with at least 1 year of continuous use was considered. Medication adherence was assessed using the proportion of days covered (PDC), calculated as the number of days with medication supply divided by the total number of days in the follow-up period. Patients with PDC < 80% were noted but included in the intention-to-treat analysis. The primary outcome was the occurrence of TKR surgery. Follow-up time was calculated from the index date to the date of TKR, death, loss to follow-up, or the end of the study period (31 December 2022), whichever occurred first.

### Statistical Analysis

Sample size calculations indicated that a minimum of 1800 patients (approximately 600 patients per group) would provide 80% power to detect a hazard ratio of 0.5 between any two groups, assuming a 2% TKR incidence in the reference group over a mean follow-up of 4 years, with a two-sided alpha level of 0.05. Descriptive statistics are presented as means ± standard deviations for continuous variables and frequencies (percentages) for categorical variables. Differences among the three groups were assessed using one-way analysis of variance (ANOVA) with Bonferroni post hoc tests for continuous variables and chi-square tests for categorical variables. The cumulative incidence of TKR was estimated using the Kaplan–Meier method, with group comparisons performed using the log-rank test. Cox proportional hazards regression was used to calculate hazard ratios (HRs) and 95% confidence intervals (CIs) for TKR, using bisphosphonate group as the reference. Both crude and adjusted HRs were calculated; the adjusted model controlled for age and comorbidities (hypertension, diabetes mellitus, dyslipidemia, liver disease, cerebrovascular disease, and coronary artery disease). The proportional hazards assumption was verified using Schoenfeld residuals. Given the baseline characteristics differences among treatment groups, inverse probability of treatment weighting (IPTW) was applied to balance covariates and calculate weighted hazard ratios (IPTW-HR) for TKR risk among the three medication groups [20]. The Covariates listed in Table 1 were used to calculate the propensity score using the Toolkit for Weighting and Analysis of Non-equivalent Groups (TWANG package in R version 9.5) [21]. To balance the covariates between the groups, the inverse probability of the weights of the propensity scores was calculated (Please refer to Appendix A). This included a generalized boosted model based on 5000 regression trees to define the weights for the optimal balance for each study group (R-gbm algorithm). Weight estimates representing the average effects of the three groups were then derived [22].

## 3. Results

A total of 6155 postmenopausal women with PTOA were included in this study. The mean T-scores were lumbar spine −2.8 ± 0.9, femoral neck −2.6 ± 0.8, and total hip −2.4 ± 0.7, with no significant differences among treatment groups (*p* = 0.156, *p* = 0.243, and *p* = 0.189, respectively). Among them, 3146 (51.1%) received bisphosphonates, 1879 (30.5%) received denosumab, and 1130 (18.4%) received raloxifene (Table 1). The median duration of anti-osteoporotic medication use was 4.8 years (3.8–6.2) for bisphosphonates, 3.9 years (3.2–5.1) for denosumab, and 5.6 years (4.1–7.3) for raloxifene, following the same pattern as follow-up duration (*p* < 0.001). The mean age was 69.42 ± 10.01 years, with significant differences across treatment groups (*p* < 0.001) (Table 1). Post hoc analysis revealed that raloxifene users were significantly younger (65.9 ± 10.18 years) compared to both bisphosphonate (69.16 ± 9.83 years) and denosumab users (71.97 ± 9.47 years), following the pattern R<B<D. Significant differences in comorbidity prevalence were also observed among the three groups (Table 1). Hypertension was more prevalent in the denosumab group (47.2%) than in the bisphosphonate (38.8%) and raloxifene (28.4%) groups (*p* < 0.001). Similarly, the incidence of diabetes mellitus was highest in the denosumab group (23.0%), followed by bisphosphonate (18.7%), and raloxifene (14.8%) groups (*p* < 0.001). Other comorbidities, including dyslipidemia, liver disease, cerebrovascular accident, and coronary artery disease, showed significant between-group differences (all *p* < 0.001). Concurrent medications and treatments for knee osteoarthritis were commonly used across all groups. NSAIDs were used intermittently by 4456 participants (72.4%), with no significant difference among treatment groups (*p* = 0.312). Regular acetaminophen use was documented in 2819 participants (45.8%), topical analgesics in 1920 participants (31.2%), and glucosamine/chondroitin supplements in 1760 participants (28.6%). Non-pharmacological treatments included physical therapy in 2394 participants (38.9%) and intra-articular corticosteroid injections in 942 participants (15.3%) during the follow-up period. Hyaluronic acid injections were administered to 535 participants (8.7%). The distribution of concurrent treatments was generally balanced across medication groups after IPTW adjustment. The mean follow-up duration was 5.47 ± 3.39 person-years. Raloxifene users had the longest follow-up period (7.31 ± 3.76 years), followed by bisphosphonate (5.63 ± 3.35 years) and denosumab users (4.10 ± 2.53 years) (*p* < 0.001), following the pattern D<B<R. During the follow-up period, 99 patients (1.6%) underwent TKR. Crude TKR rates were 2.3% in the bisphosphonate group, 0.9% in the denosumab group, and 0.9% in the raloxifene group, with a borderline significant difference among the groups (*p* = 0.052).

Cox proportional hazards regression analysis was performed to identify factors associated with TKR risk (Table 2). In the crude analysis, raloxifene was significantly associated with protective effect against TKR compared to bisphosphonates (HR: 0.62, 95% CI: 0.42–0.93, *p* = 0.022), while denosumab showed no significant association (HR: 1.11, 95% CI: 0.78–1.57, *p* = 0.557). However, after adjusting for age and comorbidities, the protective effect of raloxifene was attenuated and became non-significant (adjusted HR: 0.71, 95% CI: 0.47–1.07, *p* = 0.102). Denosumab remained non-significantly associated with TKR risk in the adjusted model (adjusted HR: 1.04, 95% CI: 0.73–1.47, *p* = 0.844). Among the comorbidities analyzed, several factors were significantly associated with TKR risk in the adjusted model (Table 2). Dyslipidemia and (adjusted HR: 1.44, 95% CI: 1.02–2.04, *p* = 0.041) liver disease (adjusted HR: 1.69, 95% CI: 1.11–2.58, *p* = 0.015) were significantly associated with increased TKR risk. Conversely, a history of cerebrovascular accident was significantly associated with a reduced TKR risk (adjusted HR: 0.22, 95% CI: 0.10–0.51, *p* < 0.001). Age was not significantly associated with TKR risk in either the crude or adjusted analyses. Other comorbidities including hypertension, diabetes mellitus, and coronary artery disease were not significantly associated with TKR risk in the final adjusted model.

To further validate our findings and address potential confounding by indication, we performed an IPTW analysis using propensity scores. The propensity scores were estimated for the average treatment effect in the entire cohort, with all covariates listed in Table 1 included as predictors. As presented in Table 3, the IPTW analysis included 6155 weighted observations and employed two methods for balance assessment: Method 1 used the mean effect size across variables, and Method 2 utilized Kolmogorov–Smirnov statistics summarized across variables with the mean. The IPTW analysis results supported our primary Cox regression findings. Using the bisphosphonate group as reference, the denosumab group showed no significant difference in TKR risk (IPTW-HR: 1.09; 95% CI: 0.88–1.35, *p* = 0.432 in Method 1; and IPTW-HR: 1.10; 95% CI: 0.89–1.36, *p* = 0.378 in Method 2). Importantly, raloxifene maintained its protective effect against TKR across both methods, with consistent IPTW-HR of 0.81 (95% CI: 0.67–0.99, *p* = 0.040) in Method 1 and 0.81 (95% CI: 0.67–0.99, *p* = 0.041) in Method 2. These findings indicate that raloxifene treatment was associated with a 19% reduction in TKR risk compared with bisphosphonate treatment after accounting for treatment selection bias through propensity score weighting. The consistency of results between the traditional Cox regression and PTW analyses strengthens the evidence for raloxifene’s protective effect against TKR in postmenopausal women with PTOA. The slightly attenuated effect size in the IPTW analysis (HR 0.81 vs. 0.29 in, adjusted Cox model) likely reflects a more conservative estimate obtained after rigorous adjustment for treatment selection bias and baseline differences among the treatment groups.

**Figure 1:**
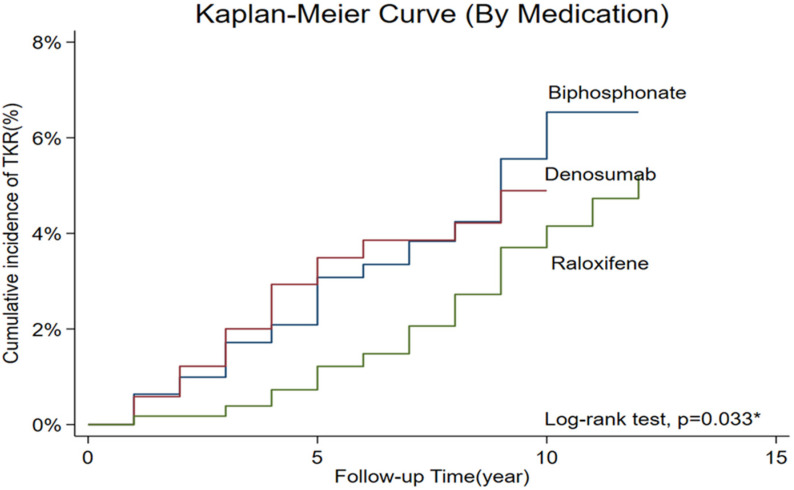
Kaplan–Meier survival curves comparing time to total knee replacement among different anti-osteoporotic treatment groups in postmenopausal women with post-traumatic osteoarthritis. * *p*-value < 0.05 was considered statistically significant.

The time-to-event analysis for TKR is illustrated in Figure 1, which presents Kaplan–Meier survival curves stratified by anti-osteoporotic medication over the entire follow-up period. The log-rank test demonstrated a statistically significant difference among the three treatment groups (*p* = 0.033). As shown in Figure 1, the bisphosphonate group had the highest cumulative incidence of TKR, reaching approximately 6.5% by the end of the follow-up period. The denosumab group showed an intermediate cumulative incidence of approximately 5%, while the raloxifene group demonstrated the lowest cumulative incidence, consistently remaining below 4%. During the first 5 years of follow-up,-survival curves remained below 2%. However, the differences became more pronounced after 5 years, with the bisphosphonate group showing a steeper increase in TKR incidence compared to the other two groups. In contrast, the raloxifene group maintained consistently lower TKR rates throughout the follow-up period, with the survival curve remaining flat, suggesting a sustained protective effect against the need for total knee replacement surgery. These Kaplan–Meier results align with the findings from both Cox regression and IPTW analyses, providing visual confirmation of the superior protective effect of raloxifene compared to bisphosphonate in preventing progression to TKR among postmenopausal women with PTOA.

## 4. Discussion

Our analysis of 6155 patients (mean follow-up: 5.47 years) found that raloxifene showed a trend toward lower TKR risk vs. bisphosphonates in crude analysis (HR 0.62, 95% CI 0.42–0.93, *p* = 0.022), but this association became non-significant in the traditional adjusted Cox model (aHR 0.71, 95% CI 0.47–1.07, *p* = 0.102). However, IPTW analysis revealed a significant protective effect of raloxifene, with consistent results across two balancing methods (IPTW-HR 0.81, 95% CI 0.67–0.99, *p* = 0.040–0.041), representing a 19% relative risk reduction. Kaplan–Meier survival analysis visually confirmed these findings: raloxifene consistently maintained the lowest cumulative TKR incidence (<4%), compared to denosumab (~5%) and bisphosphonates (~6.5%), with statistically significant differences across groups (log-rank *p* = 0.033). Denosumab showed no significant association with TKR risk in the regression analyses.

### 4.1. Raloxifene and Reduced Risk of Total Knee Replacement

#### 4.1.1. Interpreting the Raloxifene Finding and Mechanistic Plausibility

The significant TKR risk reduction with raloxifene in postmenopausal women with PTOA and osteoporosis highlights its potential clinical relevance. As an SERM, it exerts estrogenic effects that extend beyond bone density to influence bone health, mitigating cartilage degradation through TGF-β1 suppression [11], preserving type II collagen, and reducing matrix metalloproteinase activity [12], and optimizing subchondral bone metabolism and microarchitecture [11,12,14], which is crucial for osteochondral unit health [9,10], especially in estrogen deficiency, where the cartilage–subchondral bone interplay is dysregulated [23]. Given that estrogen receptor-α signaling impacts chondrocyte mechanotransduction [24], SERMs like raloxifene may support joint homeostasis via these pathways. Furthermore, the ability of raloxifene to ameliorate OA-related pain in animal models [13] may contribute to delayed TKR. Broader evidence for the role of estrogen and SERMs in mitigating OA symptoms and potentially slowing disease progression [15] lends biological plausibility to our findings.

In the specific setting of PTOA, which is often characterized by an initial acute inflammatory response and subsequent aberrant tissue remodeling, the diverse actions of raloxifene may be particularly beneficial. The development of PTOA involves a complex cascade of early molecular events following joint injury, highlighting the potential intervention [25]. The capacity of raloxifene to modulate bone turnover [14] and key signaling pathways [11] might offer a more nuanced intervention than purely antiresorptive agents in this context, especially when both OA and OP are present.

#### 4.1.2. Comparison with Existing Literature on Raloxifene/SERMs and Osteoarthritis Outcomes

Our observation of a reduced TKR risk with raloxifene in the existing literature reveals a complex and varied landscape. While direct clinical evidence specifically linking raloxifene to TKR prevention in PTOA cohorts is sparse, our results appear to resonate with preclinical work demonstrating SERM-mediated protection of joint structures [11,12,14]. It is important to acknowledge that existing clinical studies investigating SERMs and OA outcomes have presented a spectrum of results, often attributable to variations in study populations, chosen endpoints, or the type of OA under investigation. The particular focus of our study was on TKR as a definitive, hard endpoint, situated within a population where estrogen deficiency and the specific pathology of PTOA [26] are prominent factors that may offer some context for the observed association. These findings are relevant when considering the persistent challenges in developing effective disease-modifying osteoarthritis drugs (DMOADs) [26], particularly as current treatments frequently fail to halt the progression of OA [27]. Therefore, identifying existing therapeutic agents, such as raloxifene, which demonstrate promise in specific OA endotypes, potentially through strategic repurposing or advanced delivery mechanisms [24], holds considerable value. However, to move from association to causation, future research integrating molecular biomarker approaches to better delineate PTOA progression and treatment response [26] is essential, ideally through a robust framework of prospective randomized controlled trials. The temporal pattern observed in our Kaplan–Meier analysis provides additional insights into the protective mechanism of raloxifene. While all three treatment groups showed similarly low TKR rates during the first 5 years of follow-up (cumulative incidence below 2%), the protective effect of raloxifene became more pronounced after 5 years of treatment. This delayed but sustained benefit suggests that the joint-protective effects of raloxifene may require an extended treatment duration to manifest clinically, possibly reflecting the time required for its anti-inflammatory and cartilage-protective mechanisms to accumulate meaningful clinical benefits. The consistently flat survival curve for the raloxifene group throughout the observation period indicated a sustained protective effect, in contrast to the steeper increase in TKR incidence observed in the bisphosphonate group after 5 years.

Broader evidence for the role of estrogen and SERMs in mitigating OA symptoms and potentially slowing disease progression [15] lends biological plausibility to our findings. The 19% risk reduction observed in our IPTW analysis, which provided a more conservative and robust estimate after accounting for treatment selection bias, aligns with the magnitude of protection that might be expected from the comprehensive joint protective mechanisms of raloxifene. The consistency of this protective effect across the two different propensity score balancing methods (both showing an IPTW-HR of 0.81, *p* = 0.040–0.041) strengthens the evidence of a genuine therapeutic benefit. Furthermore, visual confirmation from our survival analysis showed that raloxifene maintained consistently lower TKR rates (<4%) than bisphosphonates (6.5%) throughout the follow-up period, providing compelling evidence for sustained joint protection that extends well beyond the medication’s primary osteoporosis indication.

#### 4.1.3. Time-Dependent Protective Effects and Clinical Implications

The divergence of survival curves after 5 years of follow-up observed in our Kaplan–Meier analysis suggests that the protective benefits of raloxifene against TKR may be cumulative and time-dependent. This temporal pattern has important clinical implications, as it suggests that long-term raloxifene therapy may be necessary to achieve maximal joint protective benefits in postmenopausal women with PTOA. The sustained low TKR incidence in the raloxifene group (consistently below 4%) compared with the progressive increase in the bisphosphonate group (reaching 6.5%) supports the concept that the pleiotropic effects of raloxifene on joint health extend beyond its primary indication for osteoporosis prevention. This finding aligns with the chronic, progressive nature of osteoarthritis and suggests that early initiation and sustained use of raloxifene in appropriate patients may provide long-term joint preservation benefits.

### 4.2. Clinical Context and Comparative Effectiveness

Clinical evidence for anti-resorptives in OA is mixed, and most studies have not shown consistent protection against arthroplasty. Although denosumab achieves greater BMD gains and suppresses bone turnover more than bisphosphonates, its superiority for fracture reduction is inconsistent [27,28,29,30,31,32,33]. Consistent with this uncertainty, disease-modifying effects in OA remain unclear: meta-analyses often report mixed or null bisphosphonate effects on symptoms and radiographic progression [34]. Recognizing OA as mechanistically distinct endotypes that require tailored therapy [35], injury-initiated PTOA—marked by acute inflammation and aberrant remodeling—differs from primary OA [26,36]. Within this biologically coherent subgroup of postmenopausal PTOA, our raloxifene finding provides hypothesis-generating evidence, whereas denosumab and bisphosphonates showed no clear protection in our cohort.

The relatively low TKR incidence in our cohort (1.6% over 5.47 years, or 0.29 TKR/100 person-years) compared to general aging populations may reflect the specific characteristics of our study population—postmenopausal women with post-traumatic OA receiving anti-osteoporotic therapy [37]. This selected population, combined with Taiwan’s healthcare system characteristics, cultural factors influencing surgical acceptance rates, and the inherent selection for patients with better overall bone health through active medication management, may contribute to lower TKR utilization rates compared to Western cohorts or general OA populations. These population-specific factors should be considered when interpreting our findings and their generalizability to broader clinical settings.

### 4.3. Strengths and Limitations of the Study

This study had several notable strengths. The use of real-world clinical data from a single hospital center enhances the potential applicability of our findings to similar clinical settings. The primary outcome, TKR, is a definitive and clinically significant endpoint that represents end-stage, symptomatically severe PTOA. A key strength of this study is that we focused on a clinically important and complex patient subgroup: postmenopausal women with established PTOA and concomitant osteoporosis. This allowed the exploration of treatment effects where both joint and systemic bone health are concerns. Furthermore, a direct comparison of three commonly prescribed anti-osteoporotic medications provided valuable insights, and our multivariable Cox models adjusted for several important demographic and comorbidity variables helped mitigate some potential confounding factors.

However, this study has certain limitations. Most critically, the low TKR event rate (*n* = 99, 1.6% of 6155 patients over 5.47 years) may have limited statistical power to detect modest but clinically meaningful differences, particularly for denosumab. Although IPTW analysis provided robust estimates for raloxifene, limited events resulted in wider confidence intervals and imprecision in risk estimates. The retrospective design carries inherent risks of selection bias, information bias, and unmeasured confounding despite statistical adjustments. Confounding by indication remains concerning, as anti-osteoporotic agent selection might reflect uncaptured patient characteristics or physician preferences differing between groups. The delayed raloxifene protective effect after 5 years may be influenced by differential follow-up patterns or time-varying confounders not fully accounted for. PTOA definition relied on ICD-10 codes, potentially lacking precision of prospective criteria. Critically, we lacked standardized baseline OA severity grading (Kellgren–Lawrence classification), precluding balanced severity across groups. Advanced-grade OA patients may have reduced therapy response and inherently higher TKR risk, potentially confounding results. Missing comprehensive baseline data (WOMAC scores, lifestyle factors, pain levels, functional status, conservative treatments, trauma-to-study timing) prevented adjustment for important covariates. Absence of imaging data limited the understanding of structural changes underlying TKR rate differences. Medication adherence via proportion of days covered represents indirect measurement, potentially missing actual drug exposure patterns. PTOA definition based on documented history introduced heterogeneity in injury severity, trauma timing, and affected structures, potentially influencing treatment responses. Our focus on post-traumatic osteoarthritis limits generalizability to all postmenopausal women with knee OA. PTOA’s distinct pathophysiology—younger onset and trauma-related inflammation—may produce different medication responses versus primary OA. Findings should be interpreted specifically within postmenopausal PTOA context. Additionally, this single-center Taiwan study may have limited generalizability to other ethnic groups, healthcare systems, and clinical practices. These limitations suggest our findings are informative but hypothesis-generating and require cautious interpretation. Future prospective, multicenter studies with adequate power, systematic radiographic assessment, and comprehensive baseline evaluations are warranted to establish definitive clinical recommendations.

### 4.4. Clinical Implications and Future Research Directions

Our findings suggest that raloxifene offers joint protective benefits beyond bone health in postmenopausal women with PTOA and osteoporosis, with a significantly lower TKR risk compared to bisphosphonates. Beyond the joint-protective effects demonstrated in our study, raloxifene offers additional clinical benefits, including reduced breast cancer risk, as demonstrated in the Multiple Outcomes of Raloxifene Evaluation trial [38]. This multi-benefit profile—simultaneously addressing bone health, joint preservation, and cancer prevention—further supports raloxifene’s potential as an optimal therapeutic choice for postmenopausal women with post-traumatic osteoarthritis. The combination of musculoskeletal and oncological benefits may provide additional clinical value that extends beyond the scope of our current analysis, though future studies should consider comprehensive outcome assessments to capture these multiple therapeutic benefits. For clinicians managing this complex demographic in which joint degeneration and systemic bone loss are intertwined, these results indicate that the choice of anti-osteoporotic therapy could extend beyond bone mineral density considerations alone. When multiple anti-osteoporotic options are clinically appropriate, the potential of raloxifene to favorably influence the trajectory towards TKR could be a pertinent factor in shared decision making, particularly given its established efficacy in osteoporosis management. The intermediate position of denosumab in our survival analysis coupled with the distinct response patterns observed across different anti-osteoporotic agents reinforces the concept of OA heterogeneity and the need for personalized therapeutic approaches. PTOA’s unique pathophysiology, characterized by posttraumatic inflammatory cascades and accelerated joint degeneration [26], may necessitate tailored strategies that consider both skeletal and articular health outcomes. However, individual patient factors, contraindications, and established risk-benefit profiles of each medication remain paramount in treatment selection.

Future studies should include prospective randomized controlled trials with adequate statistical power to establish the joint-protective efficacy of raloxifene in PTOA. Based on our observed effect sizes, a multicenter RCT with approximately 2000 patients and a minimum 5-year follow-up is needed to detect clinically meaningful differences in TKR incidence. Mechanistic studies elucidating raloxifene’s impact on post-injury inflammation, chondrocyte metabolism, and subchondral bone remodeling could provide insights into optimal patient selection and timing of intervention. Additionally, economic evaluations should assess the cost-effectiveness of raloxifene in preventing TKR given the substantial healthcare costs associated with joint replacement surgery.

Integration of molecular biomarkers and advanced imaging in future trials could aid in identifying patients who are most likely to benefit from early interventions, potentially even for PTOA prevention, which is a key priority highlighted by expert groups. Finally, exploring how established agents such as raloxifene may complement emerging OA therapeutics such as cell-based therapies or novel drug delivery systems represents an important avenue for advancing personalized PTOA management.

## 5. Conclusions

In this retrospective cohort study of 6155 postmenopausal women with post-traumatic knee osteoarthritis, we found that raloxifene was associated with a significant 19% reduction in TKR risk compared to bisphosphonate therapy, while denosumab showed no significant protective effect. This effect was prominent beyond 5 years of treatment and supported by complementary analytic methods. These findings suggest that the pleiotropic mechanisms of raloxifene, including estrogen receptor-mediated modulation of cartilage metabolism and subchondral bone homeostasis, may provide superior joint protection compared to purely anti-resorptive therapies in this complex patient population. When multiple anti-osteoporotic options are clinically appropriate, the potential of raloxifene to favorably influence the TKR trajectory could be a pertinent factor in shared decision-making, supporting its consideration as a potential disease-modifying therapeutic option for postmenopausal women with post-traumatic osteoarthritis and concomitant osteoporosis.

## Figures and Tables

**Table 1:** Demographics (*n* = 6155).

Females with OA	Bisphosphonates	Denosumab	Raloxifene	Total	*p*-Value	Post hoc
N	3146	1879	1130	6155		
Age, years	69.16 ± 9.83	71.97 ± 9.47	65.9 ± 10.18	69.42 ± 10.01	<0.001 *	R<B<D
Comorbidities						
HTN (%)	1222 (38.8%)	887 (47.2%)	321 (28.4%)	2430 (39.5%)	<0.001 *	
DM (%)	588 (18.7%)	433 (23.0%)	167 (14.8%)	1188 (19.3%)	<0.001 *	
Dyslipidemia (%)	884 (28.1%)	684 (36.4%)	262 (23.2%)	1830 (29.7%)	<0.001 *	
Liver disease (%)	330 (10.5%)	285 (15.2%)	95 (8.4%)	710 (11.5%)	<0.001 *	
CVA (%)	362 (11.5%)	300 (16.0%)	101 (8.9%)	763 (12.4%)	<0.001 *	
CAD (%)	412 (13.1%)	337 (17.9%)	112 (9.9%)	861 (14.0%)	<0.001 *	
Pearson-years	5.63 ± 3.35	4.10 ± 2.53	7.31 ± 3.76	5.47 ± 3.39	<0.001 *	D<B<R
TKR (%)	72 (2.3%)	17 (0.9%)	10 (0.9%)	99 (1.6%)	0.052	

Data are presented as n or mean ± standard deviation. * *p*-value < 0.05 was considered statistically significant.

**Table 2:** Factors associated with TKR (*n* = 6155).

Variables	Crude		Adjusted	
	HR (95% CI)	*p*-Value	HR (95% CI)	*p*-Value
Age	1.01 (0.99, 1.03)	0.062	1.01 (0.99, 1.03)	0.270
Medication	-	-	-	-
Bisphosphonates	Ref.		Ref.	
Denosumab	1.11 (0.78, 1.57)	0.557	1.04 (0.73, 1.47)	0.844
Raloxifene	0.62 (0.42, 0.93)	0.022 *	0.71 (0.47, 1.07)	0.102
HTN vs. None	1.52 (1.13, 2.04)	0.005 *	1.27 (0.91, 1.78)	0.162
DM vs. None	1.17 (0.81, 1.71)	0.400	0.87 (0.58, 1.30)	0.484
Dyslipidemia vs. None	1.55 (1.13, 2.11)	0.006 *	1.44 (1.02, 2.04)	0.041 *
Liver disease vs. None	2.03 (1.36, 3.03)	0.001 *	1.69 (1.11, 2.58)	0.015 *
CVA vs. None	0.29 (0.13, 0.66)	0.003 *	0.22 (0.10, 0.51)	<0.001 *
CAD vs. None	1.09 (0.70, 1.70)	0.708	0.82 (0.51, 1.3)	0.400

Data are presented as hazard ratio (95% CI) * *p*-value < 0.05, which was considered statistically significant.

**Table 3:** Risk of TKR and IPTW-HR (*n* = 6155).

	Method 1		Method 2	
	IPTW-HR (95% CI)	*p*-Value	IPTW-HR (95% CI)	*p*-Value
Medication	-	-	-	-
Bisphosphonates	Ref.		Ref.	
Denosumab	1.09 (0.88, 1.35)	0.432	1.10 (0.89, 1.36)	0.378
Raloxifene	0.81 (0.67, 0.99)	0.040 *	0.81 (0.67, 0.99)	0.041 *

Data are presented as IPTW HR (95% CI). * *p*-value < 0.05 was considered statistically significant. IPTW-HR: inverse probability of treatment-weighted hazard ratio. The propensity score was estimated as the average treatment effect in the entire cohort. The predictors of the propensity score are the covariates listed in Table 1. Method 1 uses the effect size and summarizes the variables using the mean. Method 2 uses the KS statistics and summarizes the variables using the mean.

**Figure A1:**
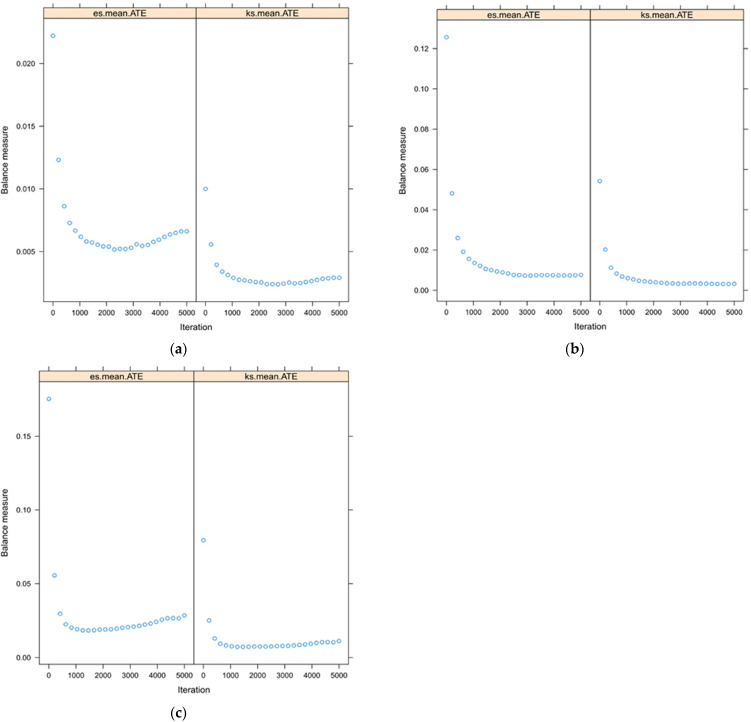
Balance measure in each iteration. (**a**) Bisphosphonate group against others. (**b**) Denosumab group against others. (**c**) Raloxifene group against others.

**Figure A2:**
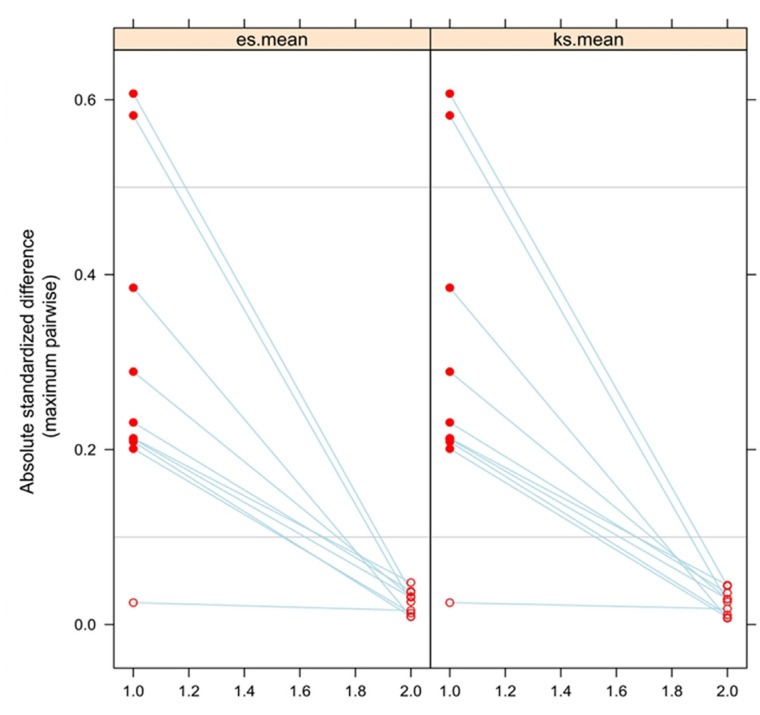
Absolute standardized difference (maximum pairwise) before and after IPTW.

## Data Availability

All generated data were within this article.

## Abbreviations

The following abbreviations are used in this manuscript:

PTOA	Post-traumatic osteoarthritis
TKR	Total knee replacement
IPTW	Inverse probability of treatment weighting
OA	Osteoarthritis
OP	Osteoporosis
SERM	Selective estrogen receptor modulators
ICD	International Classification of Diseases
PDC	Proportion of days covered
ANOVA	One-way analysis of variance
HR	Hazard Ratios

## Appendix A

**Table A1:** Balance diagnosis after propensity score weighting.

Stop Method	Max. Absolute Standardized Difference	Max. KS
Unweight	0.607	0.291
es.mean	0.048	0.019
ks.mean	0.045	0.021

The Max. absolute standardized differences were less than 0.1 in both the es.mean and ks.mean stop methods, and Max. KS also indicated that co-variates were balanced after weighting.

**Table A2:** The diagnosis of propensity score after IPTW.

Variable	Max. Absolute Standardized Difference		
	Stop Method: Unweight	Stop Method: es.mean	Stop Method: ks.mean
Age	0.607	0.037	0.044
HTN	0.385	0.010	0.007
DM	0.209	0.009	0.011
Dyslipidemia	0.289	0.032	0.029
Liver disease	0.212	0.031	0.029
CVA	0.213	0.048	0.045
CAD	0.231	0.038	0.036
HA	0.025	0.016	0.018
Tramacet	0.582	0.026	0.026
COX-2 NSAID	0.201	0.012	0.008

The Max. absolute standardized differences were less than 0.1 in both the es.mean and ks.mean stop methods, and Max. KS also indicated that co-variates were balanced after weighting.
